# An unusual rectal duplication cyst

**DOI:** 10.1186/s40792-019-0631-8

**Published:** 2019-05-09

**Authors:** Sofia Anastasiadou, Paris Tekkis, Christos Kontovounisios

**Affiliations:** 1 Department of Colorectal Surgery, Charing Cross Hospital, Chelsea and Westminster Hospital, Imperial Healthcare Trust, London, UK; 2 Department of Colorectal Surgery, Royal Marsden Hospital, London, UK; 3 Department of Surgery and Cancer, Imperial College, London, UK

**Keywords:** Rectal duplication cyst, Gastrointestinal congenital cyst, Retrorectal development cyst, Rectal mass

## Abstract

**Background:**

Rectal duplication cysts are rare gastrointestinal congenital duplicate cysts with various clinical presentations that require different management.

**Case presentation:**

We present a case of a lady with a double rectal duplicate cyst which was found incidentally on a follow-up CT abdomen and pelvis scan. The patient initially had a mucocele excision, and following that, she had a non-contrast CT abdomen and pelvis to investigate post-operative pain. The CT scan revealed a single rectal duplicate cyst. She had a posterior approach excision to have it removed, and only intra-operatively, she was found to have a double rectal duplicate cyst. She had them both removed via a midline incision running from the perineal pigmentation and extending until the coccyx. She had another follow-up CT which showed complete excision of the cysts.

**Conclusions:**

After a thorough review of the literature regarding rectal cysts, there was no mention of a double rectal duplicate cyst. The purpose of this paper is to point out the various potential presentations of a rectal cyst as well as the idea that a double cyst is managed effectively in a similar way as the single one.

## Background

Duplicate rectal cysts are the least common between the gastrointestinal congenital cysts, forming only 4% of them [1], and they are known to derive from the hindgut [2]. They are described as congenital/developmental tumours of the presacral space, and they are spherical and tubular structures, connected firmly to the intestines [3]. Although they are mostly asymptomatic, patients with duplicate rectal cysts can present with a rectal mass, constipation, pressure effects, tenesmus, or even urinary retention [4]. Patients have also presented in the past with chronic abdominal and perianal pain [5], rectal bleeding because of the ectopic gastric mucosa [4, 6], recurrent perianal abscesses [7], or rectal prolapse [4]. Clinical presentations such as colonic sub-occlusion [8], perianal sepsis [9] or even adenocarcinoma [10] have been reported in the literature as well. Rectal cysts can also co-exist with bladder exstrophy [11] or even with certain syndromes such as the Currarino syndrome [9]. Finally, there is only one mention in the literature in which the rectal cyst patient presented with a peptic ulcer eroding through the anal sphincters [12].

## Case presentation

We present the following lady with a double rectal duplication cyst which was an incidental finding. She was initially admitted because of her appendix mucocele that was resected surgically. No further intra-operative findings suggested a rectal cyst presence. However, she was still having symptoms such as lower abdominal pain and discomfort while sitting. She had a non-contrast CT scan of the abdomen and pelvis to investigate these post-operatively and to make sure there were no post-operative complications. She was, therefore, found to have a “5.7 × 2.7 cm thin-walled well-defined low attenuation structure in the right ischiorenal/ischianal fossa in close relation with the posterolateral wall of the distal rectum, extending superiorly”. This structure was crossing the levator ani and appeared to have interconnecting components but no soft tissue nodularity. These findings were consistent with a rectal duplication cyst.

**Fig. 1:**
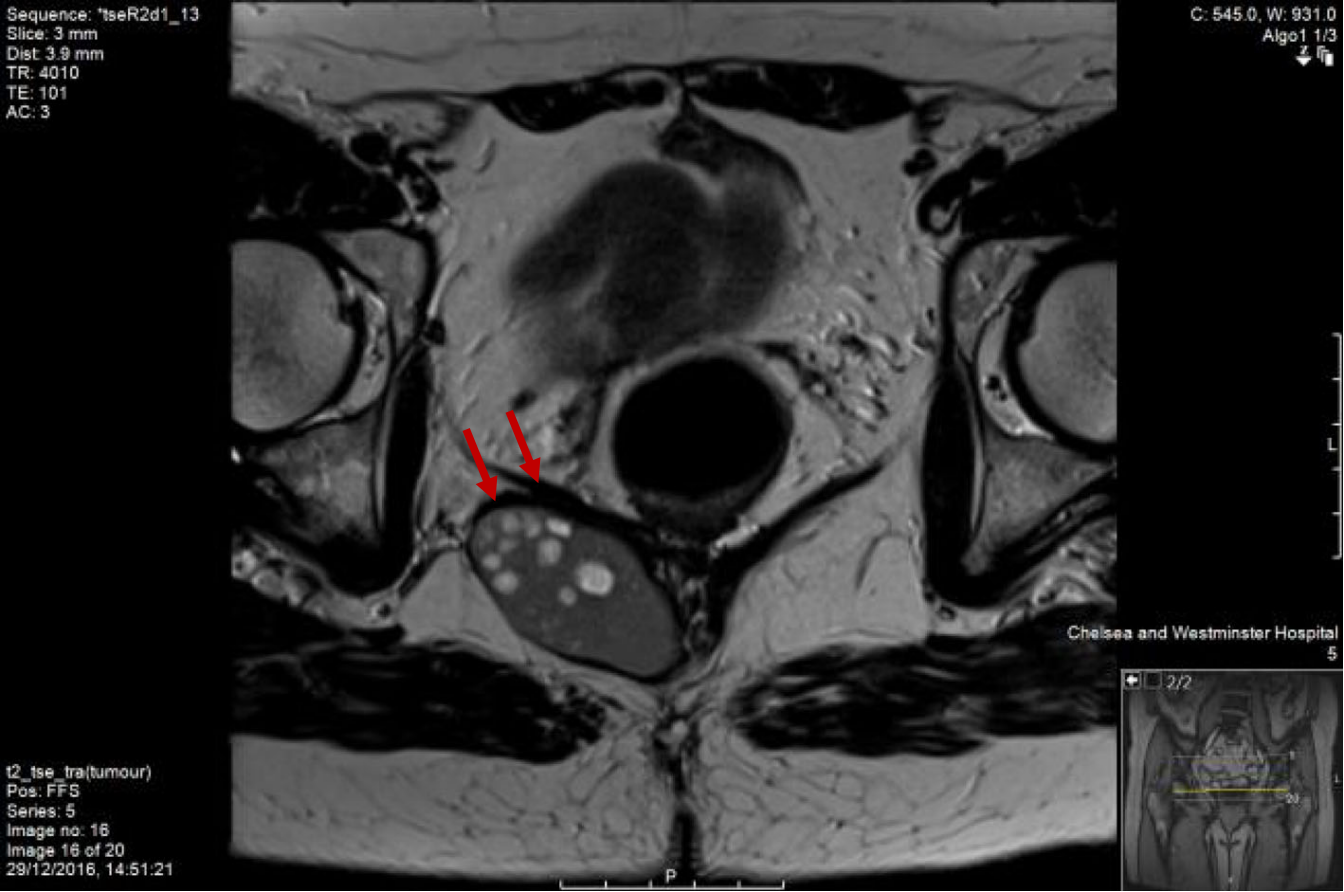
MRI scan image—transverse view

**Fig. 2:**
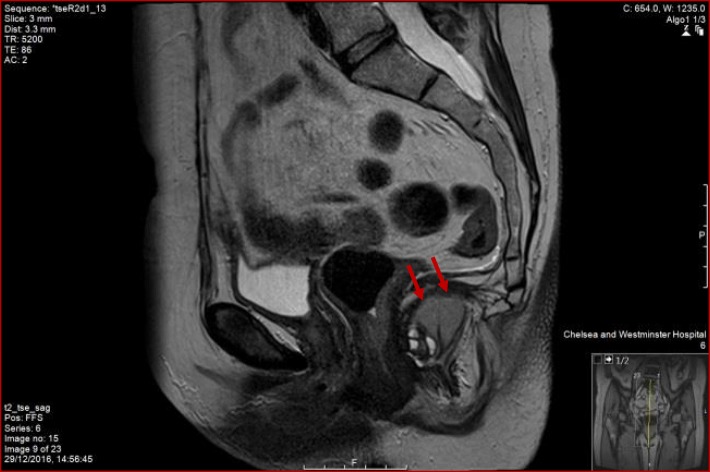
MRI scan image—sagittal view

**Fig. 3:**
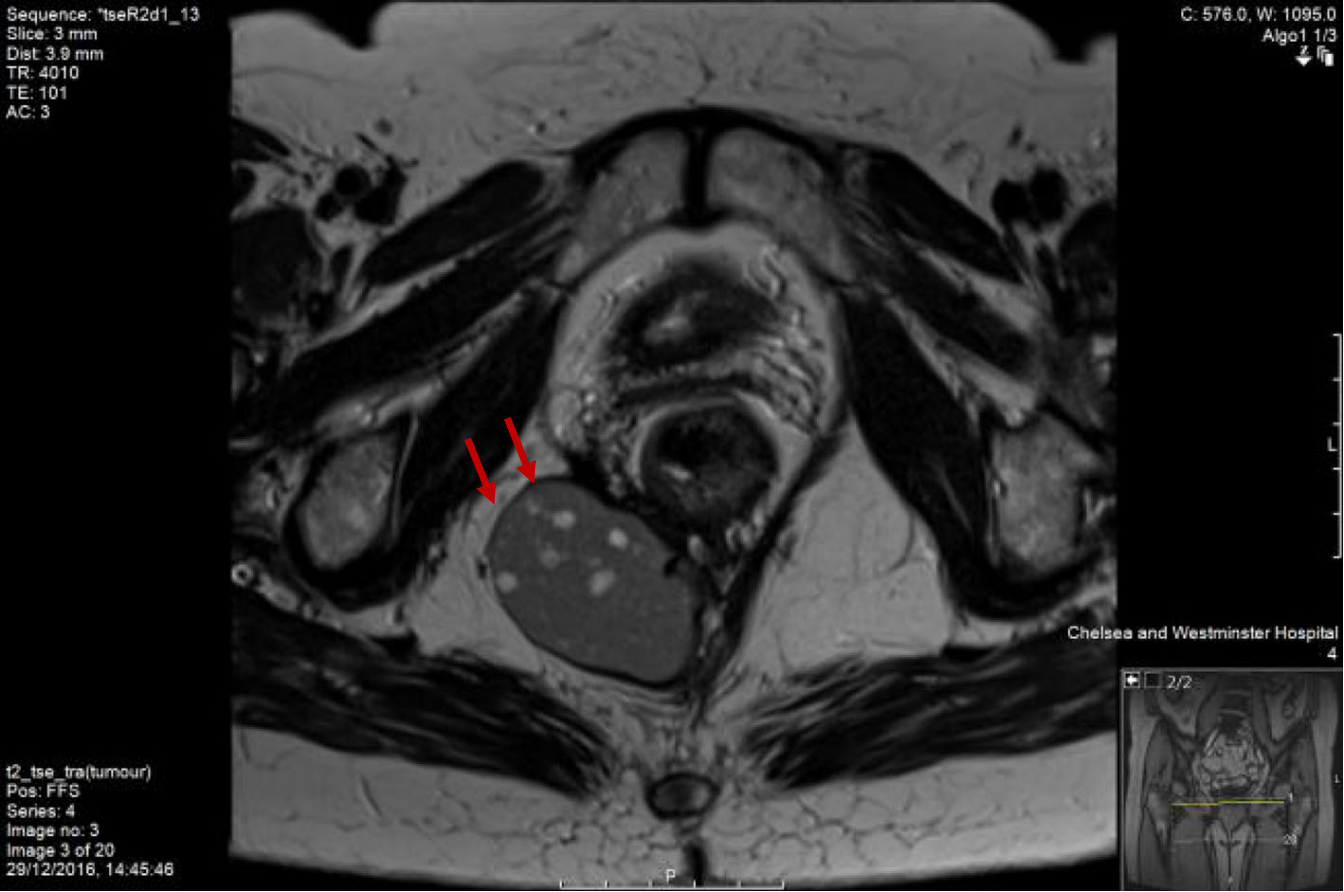
MRI scan image—transverse view

This lady was still complaining about pain while sitting down that was eventually attributed to the existence of her duplication cyst. As a result, she was investigated further and had an MRI scan (Figs. 1, 2 and 3) to specify the structure found next to her rectum. The MRI showed a rectal duplication cyst arising from the posterior fibres of the levator, extending to the left ischioanal fossa with several locules that likely contained mucinous fluid. There was no mesorectal involvement or evidence of mesorectal lymph nodes. She did not have a needle biopsy or any other investigation as the MRI did not suggest any malignant factors. She was subsequently operated to have it removed, and only intra-operatively, she was found to have a double rectal duplicate cyst. She had them both surgically removed with a posterior approach excision, were sent for histological analysis and her rectum remained intact. The procedure had no complications; however, the patient spiked temperature once post-operatively, and she was treated with antibiotics for that. She had a sepsis screen that was negative, and the episode of temperature was attributed to surgical stress. She gradually built up her diet and her pain settled as well, while her general recovery was impressively quick. She had a follow-up CT and MRI scan that both showed successful excision of the double rectal duplicate cysts while MRI showed more specifically asymmetry of the levator, but overall confirmed the previous report.

## Conclusions

Retrorectal developmental cysts are lesions that can be categorised according to their histopathological features and origins. The commonest kinds are the epidermoid cyst, dermoid cyst, rectal duplication cyst, rectal cystic hamartoma and teratoma [3]. Regarding their embryogenesis, there are two theories more dominant: (a) the Veeneklass theory that supports a malseparation of the notochord which explains the gastric epithelium and (b) the Lewis-Thyng theory that is less accepted and supports a diverticula existence on the 8–9 weeks foetus [13, 14]. Duplicate rectal cysts are the least common [1, 15]. In general, rectal cysts can be distinguished by the rest mostly due to their unique histological findings. To be more precise, rectal duplicate cysts are composed of a squamous part with a surrounding smooth-muscle element, covered with a mucus-secreting transitional epithelium. Anal ducts with apocrine secretion and lymphocyte infiltration can be also found [16]. Regarding their diagnosis, they can be found mostly with CT or MRI scan; however, a cyst was diagnosed once with plain X-ray as it was filled with a homogenous high-density substance that was radiopaque [17]. In terms of duplicate cysts’ treatment, various techniques have been attempted such as a transanal excision, a transcoccygeal, a posterior sagittal, or a combined abdominoperineal approach depending always on the positional features of the cysts [6, 18, 19]. Laparoscopic total mesorectal excision using the prolapsing technique [18] has also been tried, as well as transanal endoscopic microsurgery [20] that was completed successfully. In our case, this lady underwent a posterior approach excision with a midline incision extending from the perineal pigmentation until the coccyx, with excellent results as per her MRI scan report and her quick recovery.

A double rectal duplication cyst has never before been reported in the literature. However, clinical presentation, diagnostic tools and management are similar to the other GI duplicate cysts, and patients should be managed according to their symptomatology.

## Abbreviations

CT: Computed tomography
MRI: Magnetic resonance imaging
